# Nicotine self-administration with menthol and audiovisual cue facilitates differential packaging of CYP2A6 and cytokines/chemokines in rat plasma extracellular vesicles

**DOI:** 10.1038/s41598-021-96807-5

**Published:** 2021-08-30

**Authors:** Asit Kumar, Namita Sinha, Sanjana Haque, Sunitha Kodidela, Tengfei Wang, Angel G. Martinez, Hao Chen, Santosh Kumar

**Affiliations:** 1 Department of Pharmaceutical Sciences, College of Pharmacy, The University of Tennessee Health Science Center, Memphis, TN 38163 USA; 2 Department of Pharmacology, Addiction Science, and Toxicology, College of Medicine, The University of Tennessee Health Science Center, Memphis, TN 38163 USA

**Keywords:** Biochemistry, Molecular biology

## Abstract

In this study, we investigated whether intravenously self-administered nicotine with menthol and audiovisual cue modulates nicotine-metabolizing CYP2A6, oxidative stress modulators, and cytokines/chemokines in plasma extracellular vesicles (EVs) in rats. We assigned rats to self-administered nicotine with: (a) audiovisual cue (AV), (b) menthol, and (c) menthol and AV cue. We found increased levels of CD9 in plasma EVs after self-administered nicotine with menthol and AV cue. Moreover, expression of CYP2A6 in plasma EVs was significantly increased after self-administered nicotine in response to menthol and AV cue. However, despite an upward trend on SOD1 and catalase, increase was not found to be statistically significant, while total antioxidant capacity was found to be significantly increased in plasma and plasma EVs obtained after self-administered nicotine with menthol and AV cue. Among cytokine and chemokine profiling, we found a significant increase in the levels of MCP-1 after self-administered nicotine with menthol and AV cue and complete packaging of IL-1β in EVs. Taken together, the study provides evidence that nicotine in response to menthol and AV cues can package altered levels of CYP2A6, and cytokines/chemokines in plasma EVs that may contribute to cell–cell communication, nicotine metabolism, and inflammation upon cigarette smoking.

## Introduction

Cytochrome P450 2A6 (CYP2A6) is known to metabolize nicotine, the major constituent of tobacco, leading to the production of toxic metabolites and induction of oxidative stress that result in tissue damage and disease progression such as liver damage and lung cancer, respectively^1^. Smoking can trigger an inflammatory signaling cascade resulting in dysregulation of the cytokine and chemokine profile and an increase in the oxidative stress in part via the CYP enzymes^2,3^. In the past decade, public health effort has resulted in significant reduction in the prevalence of tobacco smoking. However, there is a dramatic rise in the consumption of alternative products that delivers nicotine, such as e-cigarettes^4^. Menthol is the most popular and preferred e-cigarette flavor among young users^5^. Prior studies have shown that menthol in tobacco and electronic cigarette facilitate the self-administration of nicotine in rats^6,7^. More specifically, Menthol has been shown to alter the pharmacokinetics of nicotine via CYP2A6 enzyme in animal models^8^. In addition, menthol and its derivatives have shown to be inhibitors of CYP2A6 that inhibit nicotine metabolism leading to enhanced nicotine plasma concentration^9^.

Although the majority of nicotine metabolism and associated effects have been shown in the liver and lungs where CYP enzymes are highly abundant, nicotine has also shown its effect in extrahepatic cells^10^. We have demonstrated that nicotine causes upregulation of expression of CYP2A6, causing an increase in reactive oxygen species (ROS) formation in extrahepatic cells, such as astrocytes and macrophages^3,11^. Also, we have reported a possible association of oxidative stress and CYP pathway with smoking-mediated increased viral load in HIV positive individuals, likely via monocytic CYP2A6^12^. In addition, we have shown that CYP2A6 is induced by ethanol and metabolizes nicotine into cotinine and other metabolites leading to a generation of ROS in U937 monocytes^13^. However, these extrahepatic cells express a much lower (< tenfold) amount of CYP enzymes including CYP2A6 compared to liver^14^. Thus, CYP2A6 as well as other modulators of oxidative stress such as antioxidant enzymes (AOEs) and inflammatory cytokines and chemokines may be circulated via plasma and taken up by extrahepatic cells. The translocation of these modulators may occur via extracellular vesicles (EVs) or more specifically exosomes, which are released from most tissues and organs and package a variety of biomolecules^15^.

EVs have been shown to play an important role in cell–cell communication by transferring various biological cargo to recipient cells^16^. Recently, we have discussed the role of EVs in packaging CYP enzymes followed by EV-mediated cell–cell interactions^17^. As we demonstrated earlier that CYP enzymes can be specifically packaged in EVs and their relative mRNA levels were found in EVs higher than in plasma^18^. In our previous study, we measured the levels of cytokines and chemokines in EVs derived from monocytic cells upon exposure to cigarette smoke condensate^19^. Also, we observed differential packaging of these agents in EVs derived from HIV-infected alcohol drinkers and cigarette smokers’ populations^20^. Therefore, in this study, we aimed to investigate the packaging of CYP2A6 and inflammatory and oxidative stress modulators in EVs released in response to nicotine and menthol using an animal model. Since an audiovisual (AV) has also been found to facilitate nicotine intake^6^, we also studied the interaction between nicotine, menthol, and an AV cue in this study.

## Results

### Characterization of EVs

**Figure 1:**
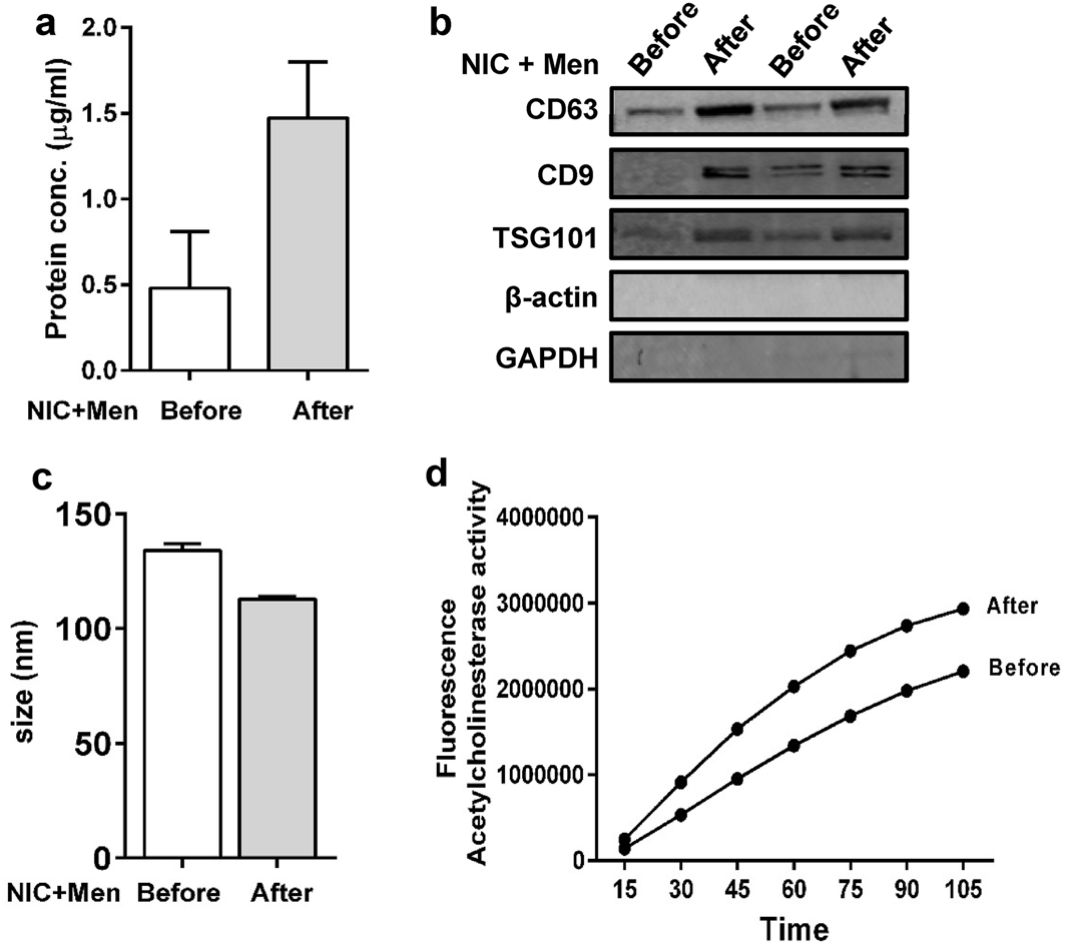
(**a**–**c**) Characterization of EVs using representative rat plasma EV samples ‘before’ and ‘after’ nicotine self-administration. (**a**) Protein quantification of rat plasma EVs obtained ‘before’ and ‘after’ self-administered nicotine with menthol cue (n = 2). (**b**) EVs were characterized using markers such as CD63, CD9, and TSG101 ‘before’ and ‘after’ self-administered nicotine with menthol cue. Cellular proteins such as β-actin and GAPDH were used as negative control (representative western blots, n = 2). (**c**) Comparison of EV size ‘before’ and ‘after’ self-administered nicotine with menthol cue (n = 2). (**d**) Acetylcholinesterase (AChE) activity was measured in rat plasma EVs. 10 µl plasma was used to isolate EVs from rat plasma. Graphs represent time-dependent curve of AChE activity of the rat plasma EVs isolated from samples ‘before’ and ‘after’ nicotine self-administration (n = 1).

In this study, we performed basic characterizations of EVs obtained from plasma ‘before’ and ‘after’ self-administration of nicotine using representative rat samples. We measured the protein concentrations in EVs isolated from rat plasma samples obtained ‘before’ and ‘after’ self-administered nicotine with menthol cue (Fig. 1a). An increased protein concentration, using limited replicates, was observed in the plasma-derived EVs obtained ‘after’ self-administered nicotine with menthol cue compared to plasma EVs obtained ‘before’ self-administered nicotine. We also confirmed the presence of the EV marker proteins CD63, CD9, and TSG101 with western blotting in the plasma EVs obtained ‘before’ and ‘after’ self-administered nicotine with menthol (Fig. 1b). The level of these proteins appear to increase after nicotine self-administration, which is consistent with an increased levels of total proteins (Fig. 1b vs. a). Further, we also confirmed the quality of EVs by measuring their size distribution, which is in the range of 100–150 nm and acetylcholine esterase (AChE) activity. The average size of EVs was similar after self-administered nicotine in the presence of menthol as compared to samples taken before nicotine self-administration (Fig. 1c). The AChE activity, which is another marker for EVs, from a representative sample was slightly higher following nicotine self-administration with menthol compared to ‘before’ self-administration (Fig. 1d). The increased AChE activity could reflect slightly higher amounts of total protein and marker proteins upon nicotine self-administration. Since these data are basic characterizations of EVs isolated from the limited representative rat samples, we do not infer the importance of the slight changes in their characteristics that we obtained.

### Effect of nicotine self-administration on EV marker protein CD9

**Figure 2:**
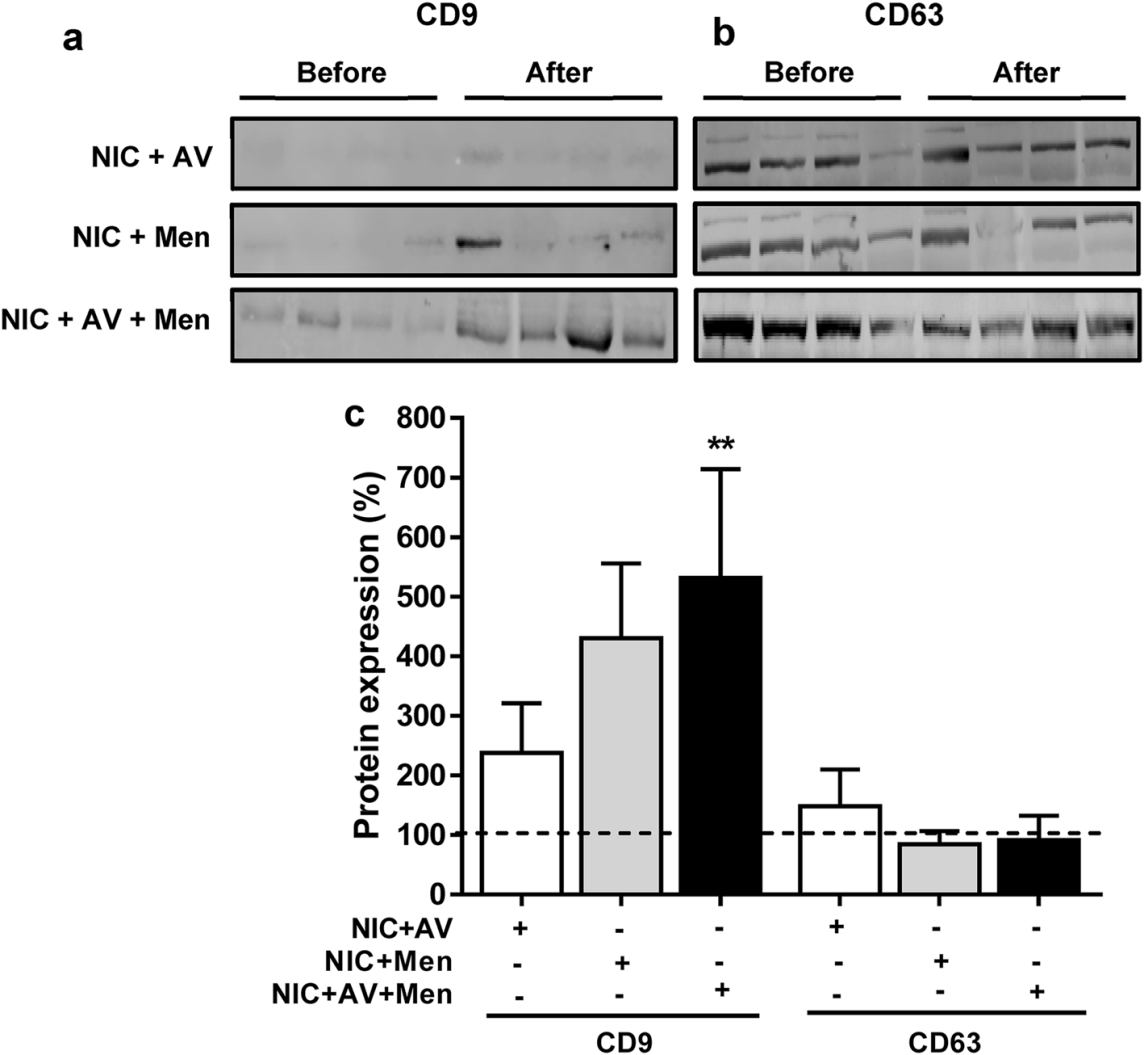
Expression of proteins in EVs. EVs were isolated from rat plasma obtained ‘before’ and ‘after’ self-administered nicotine in the presence of either AV, menthol or combined (NIC: Nicotine, AV: Audiovisual, Men: Menthol). Representative western blots of CD9 (**a**) and CD63 (**b**). The ‘before’ represent the plasma EVs obtained before the self-administered nicotine, whereas ‘after’ represent plasma EVs obtained after the self-administered nicotine with menthol and AV cue alone or combined. (**c**) Densitometry analysis. Plasma EVs obtained ‘before’ self-administered nicotine were considered as 100%. Statistical analyses were carried out by using one-way ANOVA. Results are expressed as means ± S.E.M of n = 4 experiments. **p < 0.01, in comparison with ‘before’ nicotine self-administration.

Since preliminary EV characterizations showed a marked decrease in the levels of CD9, we evaluated the expression of EV markers CD9 and CD63 to determine whether self-administered nicotine with menthol and AV cue alters the tetraspanins expression (Fig. 2). The expression of CD9 was found significantly increased (Fig. 2a,c, **p < 0.01) in rat plasma EVs obtained ‘after’ self-administered nicotine with menthol and AV cue compared to plasma EVs obtained ‘before’ self-administered nicotine. Though not statistically significant, the level of CD9 appear to be increased in plasma EVs obtained ‘after’ self-administered nicotine with both menthol and AV alone compared with their respective controls. However, no differences in the expression of CD63 were detected (Fig. 2b,c). Plasma EVs obtained ‘before’ self-administered nicotine are represented as 100% respective to their groups.

### Effect of nicotine self-administration on EV packaging of CYP2A6 and α7 nAChR

**Figure 3:**
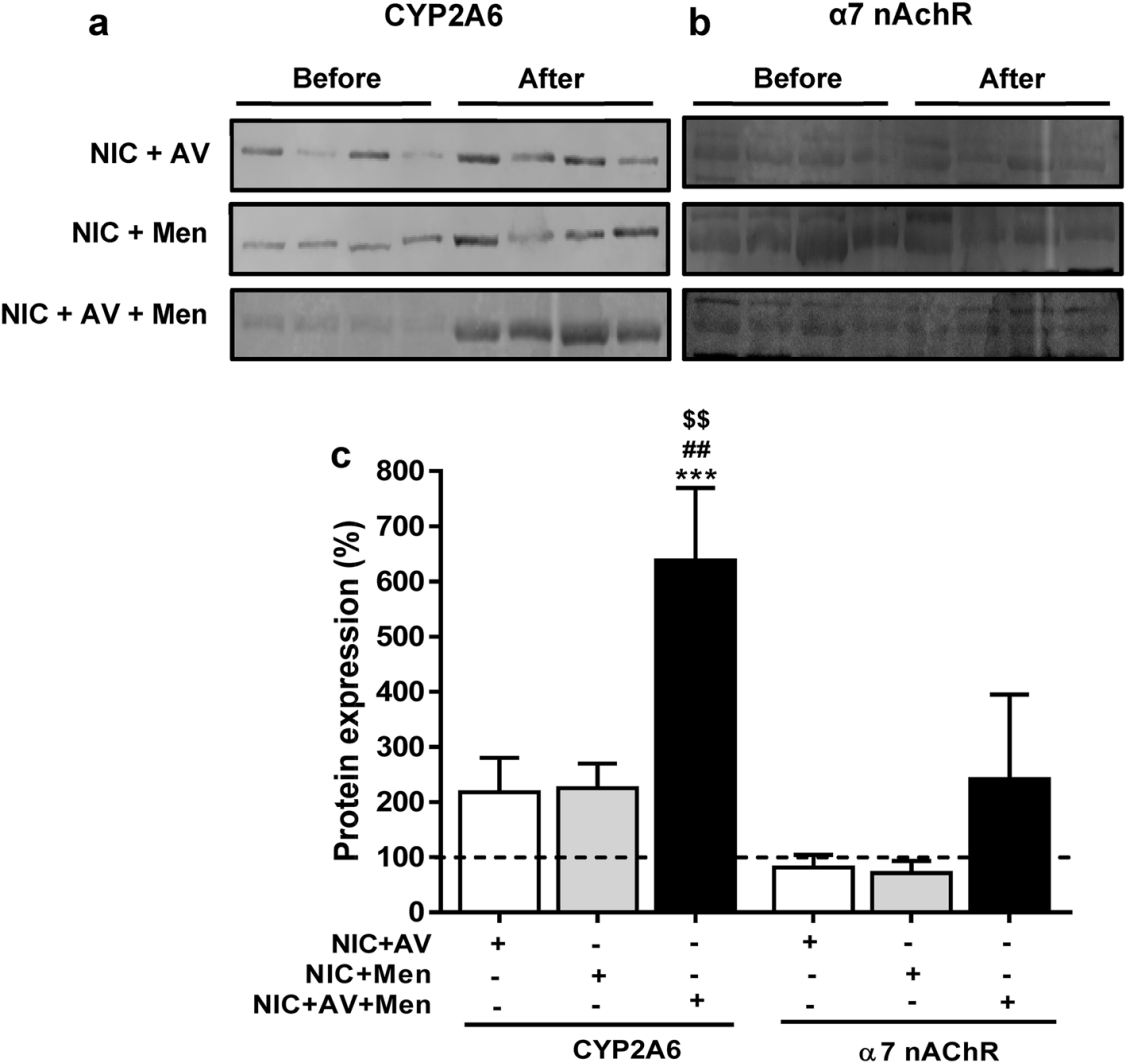
Expression of nicotine-metabolizing and nicotine receptor proteins in EVs. EVs were isolated from rat plasma obtained ‘before’ and ‘after’ self-administered nicotine in the presence of either AV, menthol or combined (NIC: Nicotine, AV: Audiovisual, Men: Menthol). Representative western blots of CYP2A6 (**a**) and α7 nAChR (**b**). The ‘before’ represent the plasma EVs obtained before the self-administered nicotine, whereas ‘after’ represent plasma EVs obtained after the self-administered nicotine with menthol and AV cue alone or combined. (**c**) Densitometry analysis. Plasma EVs obtained ‘before’ self-administered nicotine were considered as 100%. Statistical analyses were carried out by using one-way ANOVA. Results are expressed as means ± S.E.M of n = 4 experiments. ***p < 0.001, in comparison with ‘before’ nicotine self-administration. $$p < 0.01 in comparison with ‘after’ self-administered nicotine with AV. ##p < 0.01 in comparison with ‘after’ self-administered nicotine with menthol.

We hypothesized that the packaging of nicotine-metabolizing CYP2A6 enzyme and nicotine receptor α7 nAChR in plasma-derived EVs is altered upon self-administered nicotine in the presence of menthol, AV or both combined (Fig. 3). We observed that self-administered nicotine in the presence of menthol and AV cues increased the packaging of CYP2A6 in rat plasma-derived EVs (Fig. 3a,c, ***p < 0.001 in comparison with plasma EVs obtained ‘before’ nicotine self-administration). As predicted, we observed a significant increase in the packaging of CYP2A6 in combined group also as compared to self-administered nicotine in the presence of either AV (^$$^p < 0.01 in comparison with ‘after’ self-administered nicotine with AV) or menthol (^##^p < 0.01 in comparison with ‘after’ self-administered nicotine with menthol). Furthermore, the levels of α7 nAChR did not significantly changed by self-administered nicotine either in the presence of menthol, AV or both (Fig. 3b,c).

### Effect of nicotine self-administration on EV packaging of AOEs

**Figure 4:**
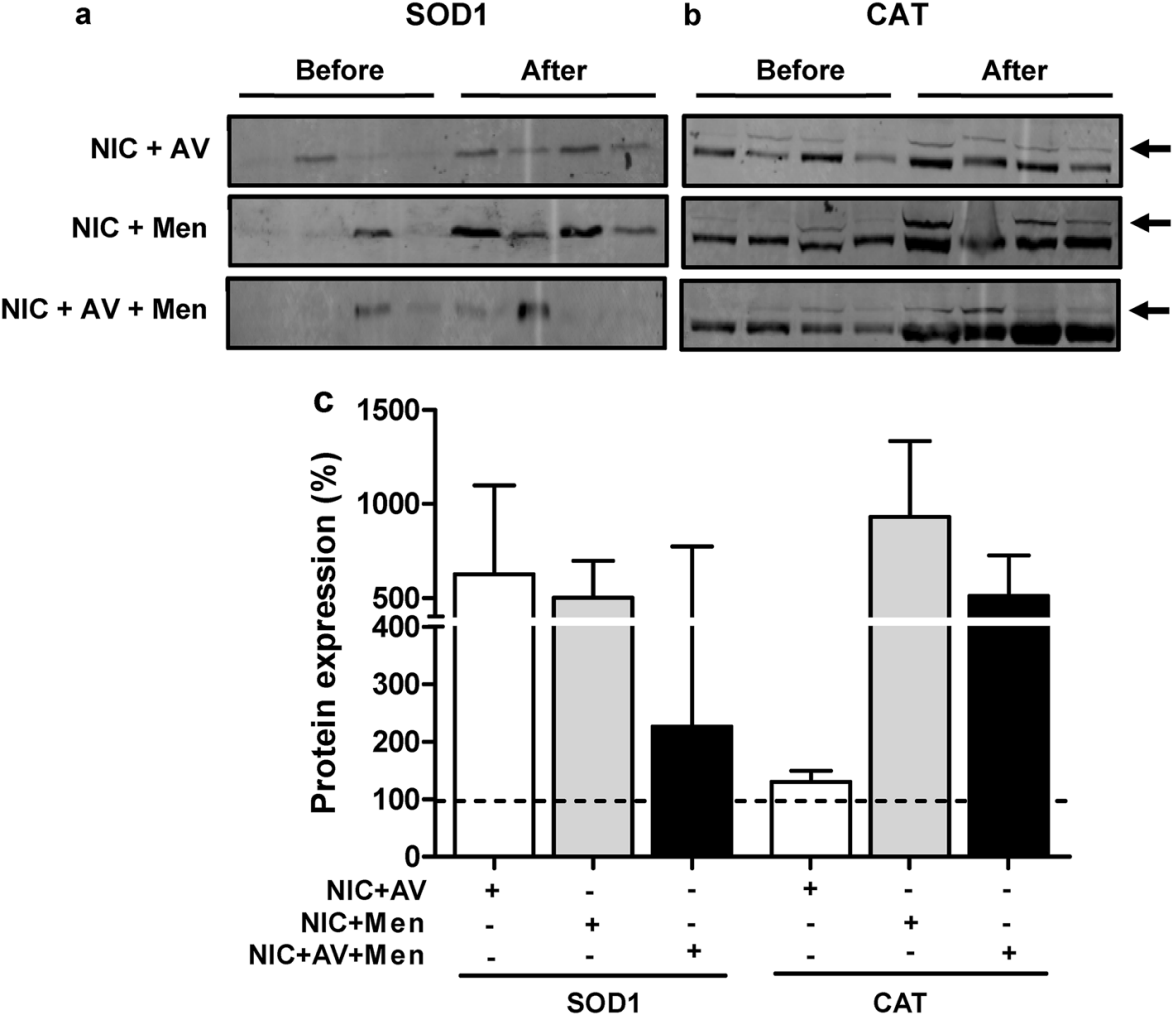
Expression of antioxidant enzymes (AOEs) in EVs. EVs were isolated from rat plasma obtained ‘before’ and ‘after’ self-administered nicotine in the presence of either AV, menthol or combined (NIC: Nicotine, AV: Audiovisual, Men: Menthol). Representative western blots of SOD1 (**a**) and CAT (**b**). The ‘before’ represent the plasma EVs obtained before the self-administered nicotine, whereas ‘after’ represent plasma EVs obtained after the self-administered nicotine with menthol and AV cue alone or combined. (**c**) Densitometry analysis. Plasma EVs obtained ‘before’ self-administered nicotine were considered as 100%. Statistical analyses were carried out by using one-way ANOVA. Results are expressed as means ± S.E.M of n = 4 experiments.

In response to oxidative stress caused by CYP2A6 mediated nicotine metabolism, cells express AOEs to neutralize the excessive accumulation of ROS generated by various stress factors. Previous studies suggested selective packaging of CYP and AOEs mRNA and protein in EVs obtained from cigarette smoke condensate (CSC) treated U937 monocytic cells compared to control^19^. Here we measured the AOEs (SOD1 and catalase) packaging, which can be altered upon nicotine metabolism-induced oxidative stress, in the plasma EVs isolated from a rat with self-administered nicotine with AV and menthol (Fig. 4). Though finding did not reach statistical significance but show an upward trend in the levels of SOD1 (Fig. 4a,c) and catalase (Fig. 4b,c) in plasma EVs obtained after self-administered nicotine with menthol and AV cue.

### Effect of nicotine self-administration on total antioxidant capacity

**Figure 5:**
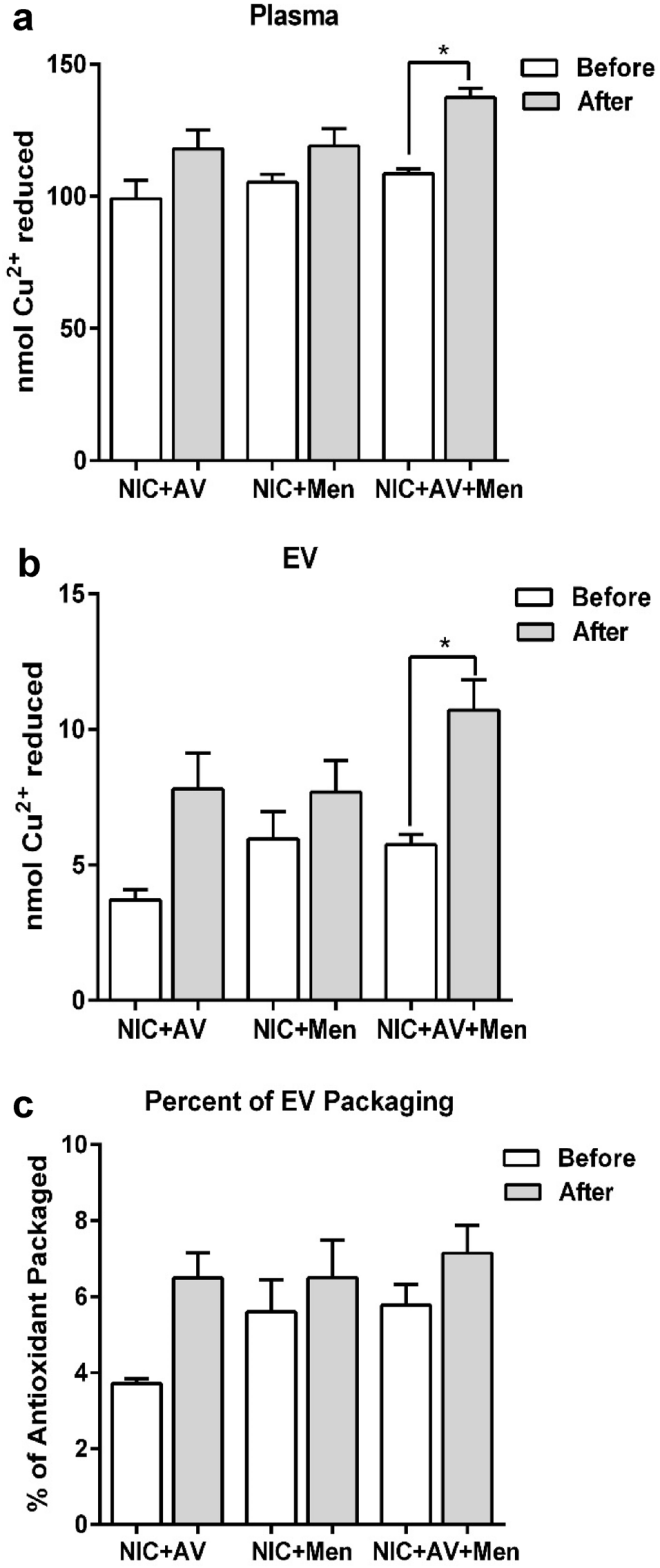
Trolox equivalent of total antioxidant capacity (TAC) in rat plasma and EVs. (**a**) Plasma obtained ‘before’ and ‘after’ self-administered nicotine in the presence of either AV, menthol or combined (NIC: Nicotine, AV: Audiovisual, Men: Menthol). (**b**) EVs were isolated from rat plasma obtained ‘before’ and ‘after’ self-administered nicotine in the presence of either AV, menthol or combined. (**c**) Percent of EV packaging. Results are expressed as means ± S.E.M of n = 3–4 experiments. *p < 0.05, in comparison with before nicotine self-administration.

Increased levels of nicotine-metabolizing CYP2A6 enzyme and AOEs suggest altered levels of the packaging of antioxidant capacity in EVs upon nicotine self-administration. Therefore, we performed the total antioxidant capacity (TAC) assay on plasma (Fig. 5a), plasma-derived EVs (Fig. 5b) and determined the relative packaging of antioxidant capacity in EVs (Fig. 5c). The results showed that compared to plasma, EVs contain 4–8% of TAC. There was also a pattern of increase in TAC upon self-administered nicotine with menthol and/or AV cue in both plasma and EVs. However, a majority did not reach statistical significance, except for self-administered nicotine with menthol and AV cue in plasma and EVs. However, the percentage of total antioxidant capacity appear to be increased in EVs (compared to plasma; relative packaging) ‘after’ self-administered nicotine with menthol and AV cue but could not reach to statistical significance compared to EVs obtained ‘before’ self-administered nicotine (Fig. 5c).

### Effect of nicotine self-administration on cytokines and chemokine

**Figure 6:**
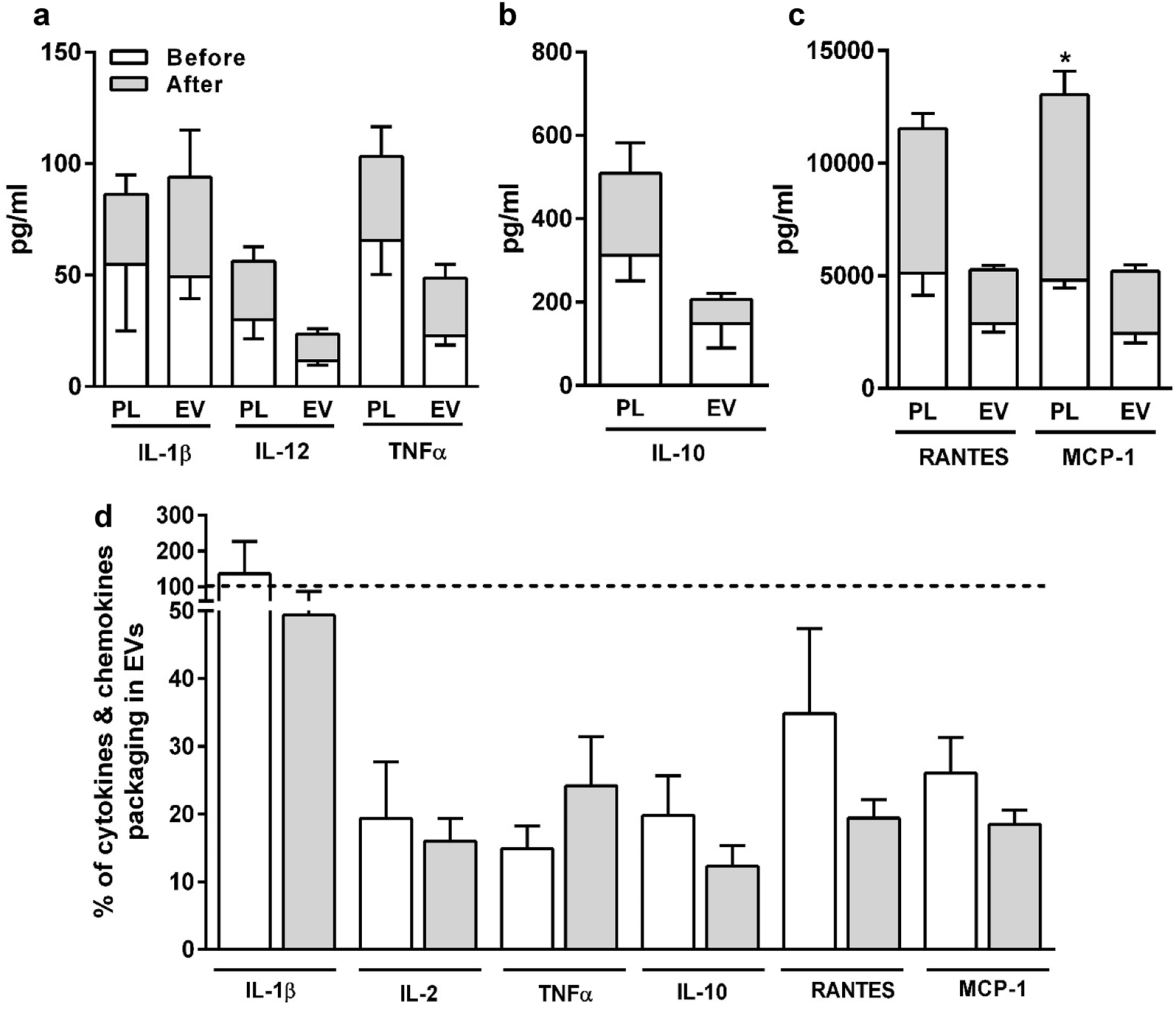
Levels of cytokines and chemokines in rat plasma and EVs. EVs were isolated from rat plasma obtained ‘before’ and ‘after’ self-administered nicotine in the presence of menthol and AV cue (NIC: Nicotine, AV: Audiovisual, Men: Menthol). (**a**–**c**) Multiplex ELISA analyses of proinflammatory cytokine (IL-1β, IL-2, TNFα), anti-inflammatory cytokine (IL-10) and chemokines (RANTES and MCP-1) were performed in plasma and EVs obtained ‘before’ and ‘after’ self-administered nicotine in the presence of either AV, menthol or combined (NIC + AV + Men). (**d**) % of cytokines and chemokines packaging in EVs. Statistical analyses were carried out by using one-way ANOVA. Results are expressed as means ± S.E.M. *p < 0.05 of n = 3–4 experiments, in comparison with plasma/EV obtained ‘before’ self-administered nicotine in respective group.

Next, we determined the levels of pro-inflammatory cytokines: IL-1β, IL-6, IL-12, TNF-α (Fig. 6a), anti-inflammatory cytokine: IL-10 (Fig. 6b), and chemokines: RANTES and MCP-1 (Fig. 6c) in plasma and EV samples ‘before’ and ‘after’ self-administered nicotine in the presence of menthol and/or AV cue. Previously we showed an altered packaging of cytokine levels in plasma and EVs derived from monocytic cells upon CSC exposure^19,21^. The cytokine and chemokine levels were largely unchanged after self-administered nicotine, both in the plasma and in EVs compared to samples obtained ‘before’ self-administered nicotine (except MCP-1 in plasma). The percentage of majority of the cytokines and chemokines packaged in EVs was in the range of 15–30%, and their relative packaging did not change upon nicotine with or without menthol and/or AV cue administration (Fig. 6d). The most interesting finding was that the packaging of IL1-β was ~ 100% in EVs, both ‘before’ and ‘after’ nicotine self-administration, suggesting that IL-1β is solely circulating in plasma via EVs. The IL-6 was non-detectable in rat plasma and EV samples of all the study groups.

## Discussion

Numerous studies have demonstrated that individuals who smoke cigarettes and/or consume nicotine are at higher risk of developing severe illnesses or worsening the existing disease condition^22–24^. Flavoring additive such as menthol promotes the use of tobacco products or electronic cigarettes likely due to its cooling sensation effect, that later becomes a potent conditioned reinforcer when it is contingently delivered with nicotine or tobacco products^6^. Prior studies suggest that flavored tobacco products, especially menthol cigarettes, may serve as starter products to regular tobacco use for youth and young adult tobacco users^25–27^. A study has found a strong correlation between the first use of tobacco and flavored tobacco product among adult tobacco users^28^. The use of menthol as a tobacco additive increased alarmingly and therefore, studies are urgently required to understand the interaction between the properties of menthol and the effect of nicotine on the cellular changes contribute to the inflammatory cascade and oxidative stress pathway. Recently, we developed a rodent model capable of elucidating the mechanism of nicotine-mediated effects on oxidative stress and inflammation in the vulnerability to smoke cigarettes during adolescence^6^. Previously we showed that nicotine-reinforced operant behavior varies significantly across a variety of isogenic strains of rats in mid-to-late adolescence^29^. The contribution of several genes to the vulnerability to cigarette-smoking has been reported, such as opioid receptors and nicotine-metabolizing CYP2A6^30^. Our findings suggest that nicotine, in the presence of menthol and AV cue, can enhance the packaging of nicotine-metabolizing CYP2A6 as well as inflammatory and oxidative stress modulators in EVs, which may potentially exacerbate smoking-mediated changes in innate immunity. This is the first study to investigate the packaging of these components in rat plasma-derived EVs, particularly upon exposure to self-administered nicotine, in the absence and presence of AV and flavor cue.

Tetraspanins are involved in the molecular organization of the cellular plasma membrane and a number of cellular functions including cell motility and migration^31–33^. The members of the tetraspanin family such as CD9 and CD63 are important for the formation and cargo recruitment of EVs. An increased expression of CD9 in EVs with self-administered nicotine, suggests an influence of nicotine on the EV formation as CD9 appears to work as an organizer of protein and lipid complexes at the EV membrane and/or plasma membrane^34,35^. Although altered levels of EVs upon exposure to cigarette smoke extract/condensate and nicotine are known, including in our own study^21,36^, a selective increase in CD9 EV marker protein is unique. Compared to CD63, CD9 appears to be expressed in relatively smaller EVs^37^, which is consistent with an overall decrease in the size of EVs (Fig. 1c), despite an overall increase in EV proteins (Fig. 1a), upon nicotine self-administration.

CYP enzymes, especially CYP2A6 are responsible for the metabolism of endogenous compounds or xenobiotics such as tobacco constituents and subsequent induction of oxidative stress in the liver and lungs^13,17^. As a result, cells present in these organs express AOEs such as SOD1 and catalase, which may be packaged in EVs and secreted in plasma circulation^21^. Studies have shown that smoking not only enhances the risk of HIV-associated complications and immunopathogenesis but also contributes to substantial morbidity and mortality in this HIV-infected population^21,38,39^. Moreover, we provided evidence of altered packaging of cytokines and chemokines in exosomes derived from the plasma of HIV infected cigarette smokers^20^. Several reports suggest that tobacco consumption, which is highly prevalent among HIV infected population, exacerbates HIV replication via the oxidative stress pathway^12,40–42^, as well as it interacts with antiretroviral drugs and potentiate HIV progression to AIDS^43^. A higher nicotine metabolite ratio reflects higher CYP2A6 activity among smokers and is associated with faster nicotine metabolism and is associated with heavier smoking and higher nicotine intake^44–46^.

Recently, we have shown that exposure to cigarette smoke condensate increases oxidative stress and HIV replication in human monocytic cells (U937 and U1)^40^. We have also shown the involvement of CYP2A6 in nicotine metabolism and oxidative stress in U937 cells^3^. These findings were further, validated the induction in HIV-infected individuals who smoke using ex vivo studies^40^. Therefore, we studied the packaging of these oxidative stress modulators in plasma EVs obtained from rats exposed to self-administered nicotine in response to menthol and AV cue. Our study demonstrated an enhanced packaging of CYP2A6 and perhaps AOEs following self-administered nicotine with AV and menthol. Prior study suggested that reduction in α7 nAChR activity increases motivation for nicotine self-administration^47^. In our study, the levels of α7 nAChR did not significantly changed by self-administered nicotine either in the presence of menthol, AV or both suggesting nicotine in the presence of menthol and AV cue act differently on the α7 nAChR. Overall, these results suggest that enhanced production of CYP2A6, possibly in the liver and lungs, upon nicotine administration, are packaged in EVs and circulated in plasma. This finding is consistent with our recent findings that cigarette smoke exposure enhances the packaging of CYP enzymes 1A1 and 1B1, which metabolize tobacco constituents, in EVs derived from both HIV-infected and uninfected monocytic cells^19,40^. However, in the same study, cigarette smoke exposure reduced the levels of AOEs. A change in AOEs levels in either direction is common upon exposure to oxidative stress agents, and it depends upon the dose and exposure time of the agents. Thus, our finding with a marked increase in the EV AOE levels upon nicotine self-administration is not surprising.

The expression of AOEs is generally induced to combat increased oxidative stress triggered by xenobiotic agents^48,49^. As one of the major defense mechanisms, the activation of a myriad of AOEs such as SOD1 and catalase protects the cells from oxidative insult^50,51^. However, if the ROS reaches a threshold level, the AOEs pathway is compromised leading to cellular death. On the other hand, a defect in the defense system may cause either a decrease or no change in the levels of these AOEs leading to the increased level of ROS. Since there is no significant increase in the protein levels of AOEs determined in the combined group of nicotine, menthol, and AV, we suggest that the levels of ROS may have reached the threshold level preventing the significant packaging of AOEs in EVs. In general, a lack of induction of most AOEs suggests their inability to counterbalance oxidative stress generated by exposure to nicotine or smoking constituents. However, it is not clear that whether nicotine-mediated oxidative stress is due to the increased level of ROS via nicotine metabolism or the inactivation of the defense mechanism such as activation of the Nrf2 pathway. Nrf2 pathway facilitates neutralization of the excessive production of ROS generated by cigarette smoking and its additives^52^. Consistent with this, our study has shown the role of Nrf2 in tobacco constituent, benzo(a)pyrene, induced oxidative stress, and resultant HIV replication via CYP pathway^53^.

Production of cytokines and chemokines is critical upon exposure to drug abuse such as smoking and during the pathogenesis of many diseases, including early and later stages of HIV infection^12,54–56^. Elevated levels of these cytokines and chemokines can be packaged into EVs and circulated via plasma, which subsequently delivered to other distant recipient cells via EV-based intercellular communication^57^. Our study has also shown differential levels of plasma circulation and packaging of various cytokines and chemokines in EVs in HIV individuals and/or tobacco smoking^20^. In particular, Kodidela et al. showed an enhanced level of MCP-1 in the plasma of HIV-positive smokers compared to HIV-positive nonsmokers, which is consistent with our findings. Our current study with nicotine self-administered rat, which demonstrated 15–30% of EV packaging of most cytokines and chemokines, is consistent with a recently published study with human subjects who smoke^20^. However, unlike our previous study with cigarette smoke condensate treatment to HIV-infected and/or uninfected monocytic cells in which the packaging of IL-6 and IL10 was increased, the current study did not show increased packaging of cytokines/chemokines in self-administered rat plasma EVs. On the other hand, unlike our previous findings with the human plasma samples or study with in vitro monocytic cells^20,21,58^, the current study showed ~ 100% packaging of IL1-β. High packaging of IL1-β in EVs, which are circulated in plasma, could have biological significance. Literature suggests the importance of IL1-β especially in neuroinflammation^59^. The expression of IL1-β in the brain cells is the hallmark of various neuronal diseases, including HIV-associated neurocognitive disorders (HAND)^60^. Thus, it’s possible that high levels of IL1-β in circulating EVs in HIV-infected smokers infiltrate into the CNS and increases neuroinflammation leading to HAND. Smoking tobacco has been mainly shown to exacerbate neuroinflammation in HIV-infected individuals leading to neuronal damage^61^.

Due to their unique ability, EVs have been studied for their pathogenic and protective role in neurodegenerative and infectious diseases including HAND^62–65^. Moreover, cigarette smoking is highly prevalent among people living with HIV^66–68^. On the other hand, adding menthol in tobacco products may enhance the addiction or negative effects of tobacco constituents^69–71^. Since menthol is known to alter nicotine pharmacokinetics^72^, it’s possible that the presence of menthol in e-cigarette further enhances CYP-mediated oxidative stress and subsequent HIV replication, especially in the CNS. Thus, it is possible that EVs packaged with oxidative stress (CYPs and AOEs) and inflammatory mediators, would infiltrate into CNS cells and enhance oxidative stress, inflammation, and subsequently disease pathogenesis including HIV replication in the brain cells. The CNS HIV reservoir is resistant to antiretroviral drugs and is known to cause neuroinflammation and neuronal damage eventually leading to declined neuropsychological and neurocognitive performance^73^. Therefore, identifying the specific role of EVs containing cytokines/chemokines, especially MCP-1 and IL1-β, and EVs containing CYPs and AOEs, as well as other EV factors when released in response to menthol added cigarettes on HIV pathogenesis and HAND, is highly desirable. Moreover, there are also several study limitations worth noting. Though a lot of parameters appear to increase in various group in the study, they could not reach to statistical significance. This could be due to small sample size as well as large variation among the replicates. The future studies need to be conducted with large sample size to achieve statistical significance.

## Materials and methods

### Animals

Adolescent Dark Agouti and Dauh salt sensitive rats (male and female, postnatal days, 29–31) (Harlan Laboratories, Madison, WI) were bred in our vivarium and were housed in a reversed 12:12 h light–dark cycle (lights on from 9:00 p.m. to 9:00 a.m.). Rats received jugular surgery between postnatal day 36 and 38 as previously described^29,74^. In this study, rats then intravenously self-administered nicotine in the presence of (a) menthol; (b) audiovisual cue; (c) menthol and audiovisual cue. Also, blood samples were collected at the time of jugular surgery or one day after the last day of self-administration. All procedures were conducted in accordance with the NIH Guidelines Concerning the Care and Use of Laboratory Animals and were approved by the Animal Care and Use Committee of the University of Tennessee Health Science Center. The study was carried out in compliance with the ARRIVE guidelines (Animal Research: Reporting of In Vivo Experiments).

### Nicotine self-administration

Nicotine self-administration was performed according to the previously published method^6,74,75^. Briefly, a sterile Micro-Renathane catheter (OD = 0.94 mm, ID = 0.58 mm, Braintree Scientific Inc., Braintree, MA) was implanted on approximately postnatal day 38. The catheter exited from the back of the animal through a polylactic acid implant as described in prior studies. Pain medication (Carprofen, 2 mg/kg) was provided immediately after surgery. The rats received no prior operant training, water or food deprivation, or social isolation before nicotine self-administration; they were group-housed according to the treatment conditions throughout the experiment. Daily 2-h nicotine self-administration sessions were conducted in operant chambers (Med Associates Inc., St. Albans, VT) equipped with two stainless steel sipping tubes containing metal ball bearings. Both sipping tubes were connected to contact lickometer controllers. Syringes containing nicotine (30 μg/kg/injection, free base, pH 7.0–7.4) and menthol were filled before each session. Polyethylene tubing connected the nicotine syringe to the jugular catheter through a swivel; the menthol was delivered near the metal ball bearings through polyethylene tubing. The solutions that included menthol (0.01% w/v, in 0.01% Tween 80) were used as a flavor cue^6^. A stock l-menthol solution (Sigma, St. Louis, MO) (100×, 0.032 M menthol) was prepared in 50% EtOH and sealed tightly. A fresh menthol working solution (1×) was prepared daily in distilled water. This concentration of menthol was used because it can unequivocally activate the thermal-sensitive lingual fibers^76^. Methohexital (5 mg/kg; JHP Pharmaceuticals, Rochester, MI), a fast-acting anesthetic, was used to verify the patency of the jugular catheters. Rats that failed this test were excluded from the analysis.

Licking on the active spout meeting a fixed-ratio 10 reinforcement schedule with a 20 s timeout simultaneously activated both syringe pumps (model PHM100 with a 15-rpm motor, Med Associates) to deliver nicotine in 0.8–1.2 s based on body weight and 60 µl menthol in 0.76 s. Therefore, licking on the active spout (hereafter referred to as active licks) delivered the menthol only when nicotine was also delivered. Licking on the inactive spout (i.e., inactive licks) had no programmed consequence. In two groups, a contingent audiovisual cue was provided. The audiovisual cue consisted of the onset of a tone generator (Malloary Sonalert, SC628, 80 dB) and a white cue light (Med Associates, ENV-221 M, with 100 mA bulb) located above the spouts for the duration of the nicotine infusion.

### Plasma EV isolation and characterization

EVs were isolated from rat plasma obtained ‘before’ and ‘after’ self-administered nicotine with menthol, AV alone or combined and characterized as per ISEV guidelines^77,78^. EVs from plasma were isolated using an exosome precipitation kit (Invitrogen), as per the manufacturer’s recommendation. Briefly, plasma was first filtered through a 0.22 μm filter to remove large vesicles (> 200 nm). Afterwards, samples were centrifuged at 2000*g* for 20 min followed by another centrifugation at 10,000*g* for 20 min to remove any cells, cell debris, and large vesicles, presumably corresponding to the population of shedding vesicles. The supernatant was transferred to a new tube and 0.5 volumes of PBS was added to it, followed by the addition of 0.2 volumes of exosome precipitation reagent. The reaction mixture was incubated for 10 min at room temperature. After incubation, the sample was centrifuged at 10,000*g* for 10 min, which resulted desired EV pellet. The size of EVs was evaluated on Zetasizer Nano-ZS (Malvern Instruments Inc, Malvern, UK) using dynamic light scattering technique. Next, we ensured the presence of EVs by analyzing the expression of the EV marker proteins CD63, CD9, and/or TSG101 by western blotting, by loading equal amounts of protein. We also quantified and validated the EVs by measuring acetylcholinesterase (AChE) activity using the fluorescent Amplex^®^ Red Acetylcholine/Acetylcholinesterase Assay Kit (Molecular Probes, Invitrogen) as described previously^20^. Since plasma EVs have been extensively characterized, we used 1–2 representative rat samples to characterize EVs obtained from rat plasma and ensured that they have general characteristics of plasma EVs.

### Western blotting

The protein concentrations of EV samples were quantified using the Pierce BCA Protein Assay Kit (Thermo Scientific). Then, 25 µg of protein from each sample was loaded onto 10% sodium dodecyl sulfate–polyacrylamide acrylamide gel and the rest of the procedure was performed as described previously^21,58^. We evaluated the expression of proteins associated with EVs (CD63, CD9, TSG101), nicotine metabolizing enzyme (CYP2A6), antioxidant enzymes (SOD-1 and catalase), and nicotinic acetylcholine receptor (nAChR) and quantified by western blot. Following electrophoresis, proteins were transferred onto polyvinylidene difluoride membrane and blocked in Li-Cor blocking buffer (LI-COR Biosciences, Lincoln, NE) for 1 h. After blocking, membranes were incubated overnight at 4 °C with primary antibodies, including CD63 rabbit mAb (1:300 dilution, catalog no. 25682-1-AP, Proteintech), CD9 mouse mAb (1:100 dilution, catalog no. 60232-1-1g, Proteintech), TSG101 mouse mAb (1:100 dilution, catalog no. sc-7964, Santa Cruz Biotechnology), β-Actin mouse mAb (1:1000 dilution, catalog no. 3700, Cell Signaling), GAPDH rabbit mAb (1:1000 dilution, catalog no. 2118, Cell Signaling), CYP2A6 rabbit mAb (1:200 dilution, catalog no. ab3570, Abcam), nAChR rabbit mAb (1:200 dilution, catalog no. ab208641, Abcam), SOD1 mouse mAb (1:200 dilution, catalog no. sc-101523, Santa Cruz Biotechnology), and Catalase mouse mAb (1:100 dilution, catalog no. sc-365738, Santa Cruz Biotechnology). On the next day, following washing (three times for 5 min each in PBS containing 0.2% Tween 20), membranes were incubated with either goat anti-mouse or goat anti-rabbit secondary antibody (1:10,000 dilution, LI-COR Biosciences) for 1 h at room temperature in the dark. After 1 h membranes were washed and scanned using the Li-Cor Scanner (LI-COR Biosciences) (Supplementary Information). Densitometry analyses of the proteins were performed using LI-COR Image Studio Software (v.5.2, Nebraska, USA).

### Total antioxidant capacity assay

The trolox equivalent total antioxidant capacity (TAC) was determined in EVs using the commercial assay kit (Biovision, Milpitas, CA) upon following the manufacturer’s instructions. Results are expressed as µmol of Trolox equivalents (TE). All assays were done in triplicate.

### Multiplex ELISA

Levels of cytokines and chemokines such as pro-inflammatory: TNF-α, IL-1β, IL-6, IL-2; anti-inflammatory: IL-10, IL-13; and chemokines: MCP-1 and RANTES in plasma and EVs were measured using customized rat 8-Plex ProcartaPlex™ multiplex immunoassay (ThermoFisher Scientific, Waltham, MA). EVs pellet were resuspended in 50 μl universal assay buffer, and 50 μl plasma were taken directly for the cytokine analysis according to the manufacturer’s instructions. Briefly, the samples, standards, and magnetic beads were added to the 96-well ELISA plate and incubated for 1 h at room temperature. After 1 h, wells were washed and subsequently detection antibody, streptavidin-PE, and reading buffer was added followed by data acquisition. Samples were analyzed according to the operation manual of the Luminex MAGPIX^®^ system. Concentration of cytokines and chemokines were expressed as pg/ml. Next, we analyzed the relative packaging of cytokines/chemokines in EVs compared with cytokines/chemokine present in the plasma from which the EVs originated. We calculated the percentage using the following equation:

$${\text{Percent }}\,{\text{of}}\,{\text{ cytokines}}/{\text{chemokines }}\,{\text{packaging }}\,{\text{in}}\,{\text{ EVs }} = \, \left( {{\text{Concentration }}\,{\text{of}}\,{\text{ cytokines}}/{\text{chemokines}}\,{\text{ in}}\,{\text{ the}}\,{\text{ EVs }} \div {\text{ Concentration }}\,{\text{of }}\,{\text{cytokines}}/{\text{chemokines}}\,{\text{ in}}\,{\text{ the}}\,{\text{ plasma}}} \right) \, \times { 1}00.$$

### Statistical analysis

Mean ± SEM was calculated and compared to the respective control group. One-way ANOVA with Tukey’s multiple comparison test was used to calculate the statistical significance. The data were analyzed using Graph-Pad Prism version 7.0 (GraphPad Software, La Jolla, CA, USA). A p-value of ≤ 0.05 was considered statistically significant.

## Supplementary Information

Supplementary Figures.
