# Supplementary material for: Nicotine self-administration with menthol and audiovisual cue facilitates differential packaging of CYP2A6 and cytokines/chemokines in rat plasma extracellular vesicles
Source: Sci Rep. 2021 Aug 30;11:17393. doi: 10.1038/s41598-021-96807-5 (PMC8405708; doi:10.1038/s41598-021-96807-5)
Supplement: Supplementary file 1 — Supplementary Figures. [file 41598_2021_96807_MOESM1_ESM.docx]

**Supplementary Information**

**Nicotine self-administration with menthol and audiovisual cues facilitates differential packaging of CYP2A6 and cytokines/chemokines in rat plasma extracellular vesicles**

Asit Kumar ^1^, Namita Sinha^1^, Sanjana Haque^1^, Sunitha Kodidela^1^, Tengfei Wang^2^, Angel G. Martinez^2^, Hao Chen^2^, and Santosh Kumar^1,*^

^1^ Department of Pharmaceutical Sciences, College of Pharmacy, The University of Tennessee Health Science Center, Memphis, TN 38163, USA

^2^ Department of Pharmacology, Addiction Science, and Toxicology, College of Pharmacy, The University of Tennessee Health Science Center, Memphis, TN 38163, USA

*Correspondence: Santosh Kumar, [ksantosh@uthsc.edu](mailto:ksantosh@uthsc.edu)

**Fig. 1b. Original blots**


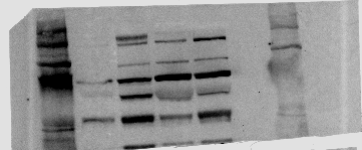


**CD63**


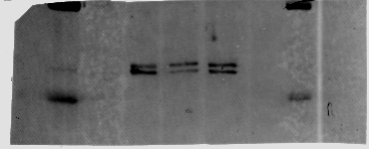


**CD9**


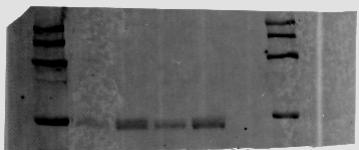


**TSG101**


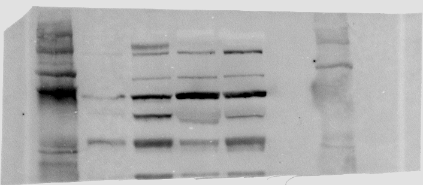


**Actin**


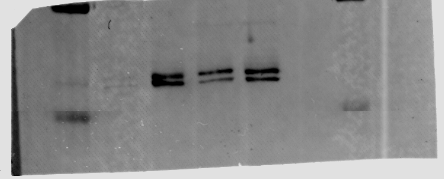


**GAPDH**

**Fig. 2a, 2b. Original blots**


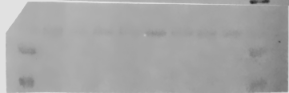


**CD9**

**NIC + AV**


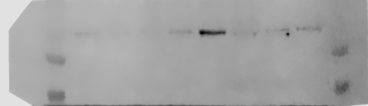


**CD9**

**NIC+ Men**


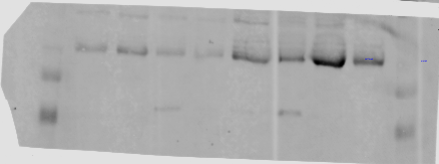


**CD9**

**NIC + AV + Men**

**CD63**


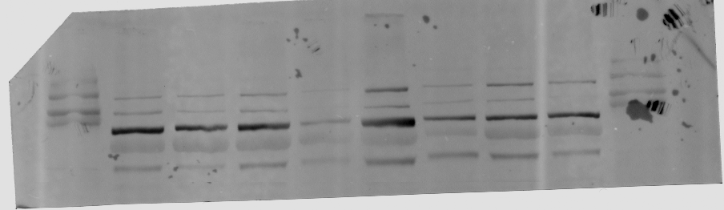


**NIC + AV**


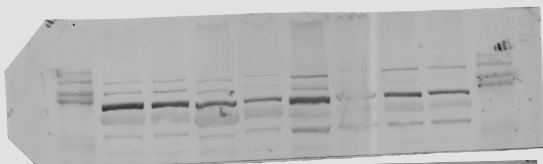


**CD63**

**NIC + Men**


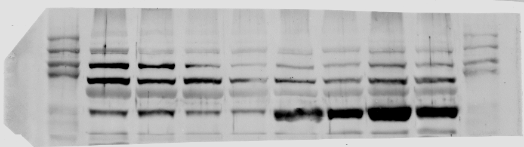


**CD63**

**NIC + AV + Men**

**Fig. 3a, 3b. Original blots**


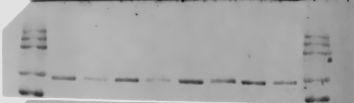


**CYP2A6**

**NIC + AV**

**CYP2A6**


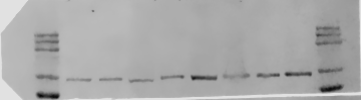


**NIC + Men**


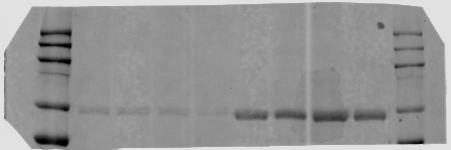


**CYP2A6**

**NIC + AV + Men**


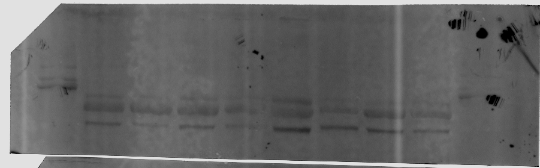


**α7 nAchR**

**NIC + AV**


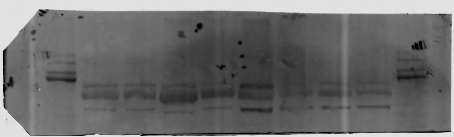


**α7 nAchR**

**NIC + Men**

**α7 nAchR**


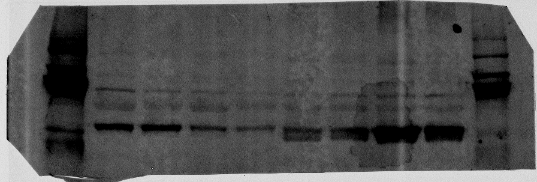


**NIC + AV + Men**

**Fig. 4a, 4b. Original blots**


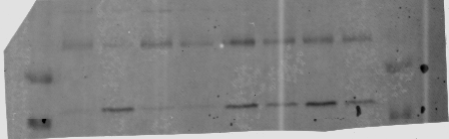


**SOD1**

**NIC + AV**


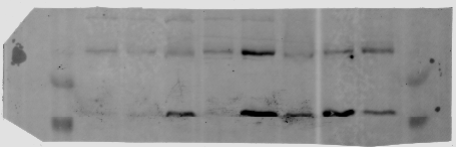


**SOD1**

**NIC + Men**


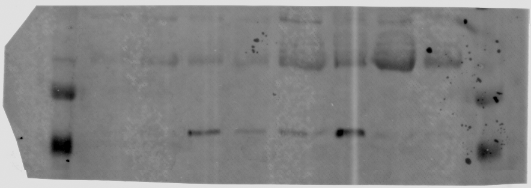


**SOD1**

**NIC + AV + Men**


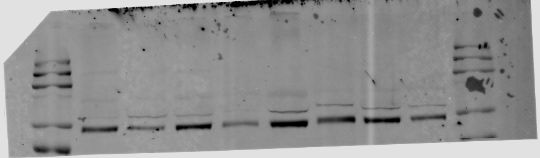


**CAT**

**NIC + AV**


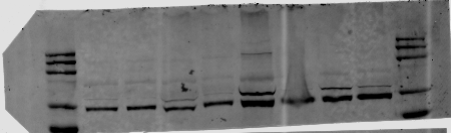


**CAT**

**NIC + Men**


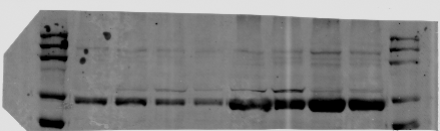


**CAT**

**NIC + AV + Men**
